# Analyzing Test Items Using Difficulty Index and Discrimination Index for Evaluating Postgraduate Medical Residents

**DOI:** 10.7759/cureus.116220

**Published:** 2026-09-14

**Authors:** Bijoy Kumer Paul, Khandakar Fatema, Taslima Chowdhury, Kamrun Nahar, Jannatul Saki, Marium Salwa, M Atiqul Haque

**Affiliations:** 1 Department of Public Health and Informatics, Bangladesh Medical University, Dhaka, BGD

**Keywords:** assessment, bangladesh, difficulty index, discrimination index, medical education

## Abstract

Background

This study aimed to analyze objective questions used to evaluate Phase B residents in the Basics of Medical Education course in Bangladesh, focusing on standardizing test questions and identifying areas for improvement based on Difficulty Index (DIF I) and Discrimination Index (DI).

Methodology

In November 2022, 148 Phase B residents were examined by an internal assessment on the Basics of Medical Education. The assessment comprised 50 objective questions, including true/false (T/F) statements and fill-in-the-gap exercises. Each correct answer received one mark. Following the assessment, item analysis was performed for validation, with data analysis conducted using Microsoft Excel (Microsoft Corp., Redmond, WA).

Results

Out of 30 T/F statements, 40% were considered good/acceptable, with a mean DIF I of 76.17 and a standard deviation (SD) of 20.42; 60% were considered too easy. The overall mean (SD) DI for all T/F statements was 0.18 (0.13), with half displaying acceptable to excellent discriminatory capacity. For the 20 fill-in-the-blank statements, 40% were considered acceptable, with a mean (SD) DIF I of 64.80 (20.70) and a DI of 0.32 (0.18), indicating that 75% of these items had a good to excellent quality level.

Conclusion

Fill-in-the-blank statements demonstrated exceptional discriminatory capabilities, but half of the T/F statements require significant refinement to enhance their quality.

## Introduction

Assessment is crucial for successful teaching and learning processes [1]. It helps identify the strengths and weaknesses of teaching approaches and academic curricula. Assessments are conducted during or after educational sessions, aiming to improve the learning journey and support effective teaching techniques [2]. Educators conduct various evaluations and tests throughout the academic calendar, with tests and examinations holding significant significance within the educational domain [3].

Assessments have important parts such as content and construct validity, reliability, and objectivity. These aspects can be evaluated well with advanced thinking skills, but it is important to know that each assessment method has its strengths and limits [4]. After the examination, item analysis examines the performance of individual items within the test by comparing them to an external standard or in relation to other items [5]. Item analysis entails conducting statistical examinations of students' test responses. By gathering and summarizing these responses, objective quantitative data can be obtained, aiding in the assessment of the quality of test items and the overall enhancement of the assessment's effectiveness [6]. Recent item-analysis studies across diverse medical education settings confirm that systematically calculating difficulty and discrimination indices, together with distractor efficiency, reliably differentiates well-functioning items from those requiring revision, reinforcing item analysis as an essential post-assessment quality-assurance step [7,8].

At Bangladesh Medical University (BMU), the use of assessment tools such as multiple-choice questions (MCQs) and short-answer questions (SAQs) in postgraduate medical examinations has been adopted due to their proven effectiveness, reliability, and ability to ensure standardized assessments. The quality of test items plays a direct role in evaluating the competency of the students. Item-analysis evidence specific to postgraduate and formative assessment settings remains comparatively limited: in a postgraduate pediatric examination, 58.3% of items showed poor discriminative ability [9], while in an obstetrics-and-gynecology formative assessment, 64% of items were excessively difficult even though most discriminated adequately [10], together underscoring how easily items can drift outside acceptable ranges without setting-specific, routine item analysis of the kind undertaken at BMU.

Educational models such as Bloom's taxonomy and Miller's pyramid emphasize that well-constructed questions possess the capacity to gauge higher-order cognitive skills such as interpretation, analysis, critical thinking, application, and synthesis [11]. Empirical work grounded in these frameworks supports their practical relevance: Thompson and Lake found that students taking a deeper learning approach performed better on both higher- and lower-order examination questions, indicating that Bloom's cognitive levels meaningfully differentiate item demands [12], while a randomized controlled trial of Bloom- and Miller-aligned e-learning modules showed significantly better cognitive, psychomotor, and affective outcomes than conventional teaching (P < 0.001) [13], reinforcing the rationale for evaluating the psychometric properties of individual items rather than relying on total test scores alone.

Beyond these theoretical frameworks, a growing body of empirical literature has examined the psychometric behavior of test items across disciplines and formats. Distractor efficiency appears closely linked to item difficulty, with items containing more functioning distractors tending to be harder [7,8]. Evidence on the distractor-efficiency-to-discrimination relationship is more mixed: Chauhan et al. found that discrimination improved as the number of functioning distractors increased (index rising from 0.23 to 0.51 across one to three distractors) [8], whereas Rezigalla et al. reported a weak negative correlation between distractor efficiency and discrimination (r = -0.259; p = 0.048) [7]. Question format itself appears to influence discriminatory power, with some evidence suggesting that constructed-response and short-answer items can outperform conventional multiple-choice items on discrimination, even though multiple-choice and true/false (T/F) formats remain the most feasible for large cohorts [14]. However, the great majority of published item-analysis studies have focused on single-best-answer multiple-choice questions [15-19]; comparatively few have examined true/false statements or fill-in-the-blank items, despite their continued use in formative postgraduate assessment because they are quick to construct and score [20,21]. This relative scarcity of literature on non-MCQ item formats, combined with the paucity of item-analysis data from postgraduate medical education programs in Bangladesh, defines the specific gap this study seeks to address.

Although difficulties are associated with developing test items, academic institutions managing assessments can gain advantages from a meticulously maintained collection of good test items facilitated by thorough item analysis. While item analysis of multiple-choice questions has been widely studied, comparative evidence on the psychometric performance of true/false and fill-in-the-blank items, both commonly used in postgraduate medical assessment, remains limited, particularly in resource-limited settings. Understanding how these two formats differ in difficulty and discriminatory power can guide test developers in selecting and refining item types best suited for evaluating postgraduate residents. This study addresses this gap by directly comparing the difficulty and discrimination indices of T/F and fill-in-the-blank items within a single postgraduate cohort in Bangladesh. This research aims to assess the quality of objective test items, rectify any inaccurately framed questions, and ultimately foster an "optimal" question bank. Moreover, this undertaking will offer valuable support to faculty members in enhancing the formulation of test items for forthcoming assessments.

## Materials and methods

This study employed a cross-sectional approach, focusing on Phase B residents of Bangladesh Medical University (BMU) (formerly Bangabandhu Sheikh Mujib Medical University {BSMMU}) residency program preparing for the "Short Course on Basics of Medical Education" examination. The study received approval from the Institutional Review Board (IRB) of Bangladesh Medical University (BMU), Shahbag, Bangladesh (memo number BSMMU/2021/4699, approved 15 July 2021), and obtained verbal consent from the participants.

The BMU residency program started in 2009. The program has two phases: Phase A and Phase B. All Phase B residents must take the "Short Course on Basics of Medical Education." This course lasts for one month and is mainly lecture-based. There are 12 interactive lectures in total. Each lecture lasts for two hours. At the end of the course, there is a written test with objective-type questions. In total, 148 residents were evaluated through an internal assessment; no formal exclusion criteria were applied, and all residents present for the assessment (n = 148) were included. In November 2022, all 148 Phase B residents enrolled in the course during the study period were included, representing a census of the eligible cohort rather than a predetermined sample. In this study, medical educationists from various disciplines, both internal and external, were invited to conduct sessions for this course. At the end of each block, facilitators were requested to submit 10 questions. Then, the course coordinator selected items based on content accuracy and the analysis of the question bank. Content accuracy was done by preparing the test matrix. Finally, the chairman's office formatted the newly structured questionnaire for the examination.

The assessment comprised 30 true or false (T/F) statements and 20 fill-in-the-gap statements, all presented as single sentences. Participants were allotted 60 minutes to finish the test. Each accurate response was awarded a score of 1, and no marks were deducted for incorrect answers. Thus, the test had the highest achievable score of 50 and the lowest score of 0.

After the assessment, all the residents' responses were recorded, and the quality of the questions was analyzed using these responses. Scores for specific questions were carefully logged for each student. Students were ranked from the highest to the lowest using these cumulative scores. The top 40 students were put in the High-Ability Group (HAG), while the bottom 40 formed the Low-Ability Group (LAG) based on their rankings.

After acquiring the necessary data and information on all the questions within the formative assessment, we evaluated the Difficulty Index (DIF I), which measures the level of difficulty experienced by all examinees. Additionally, we analyzed the Discrimination Index (DI), assessing the questions' ability to differentiate between individuals with higher and lower levels of knowledge.

Difficulty Index (DIF I) was determined by employing the following formula, which involved computing the percentage of students who provided the correct response to the item [15,20]: DIF I = [(HAG + LAG)/N] × 100, where HAG = the number of correct responses in the upper group (27% of total responses), LAG = the number of correct responses in the lower group (27% of total responses), and N = the total number of responses in both groups (Table 1).

**Table 1:** Difficulty Index interpretation

Value	Interpretation
<30%	Very difficult
30%-70%	Good/acceptable/average
>70%	Very easy

Discrimination Index (DI) was computed using the following formula, reflecting an item's capacity to distinguish between high-performing students and those with lower performance [15,20]: DI = 2 × [(HAG - LAG)/N], where HAG = the number of correct responses in the upper group (27% of total responses), LAG = the number of correct responses in the lower group (27% of total responses), and N = the total number of responses in both groups.

The Discrimination Index was interpreted in Table 2.

**Table 2:** Discrimination Index interpretation

Value	Interpretation
<0.20	Poor/revise
0.20-0.24	Acceptable
0.25-0.34	Good
≥0.35	Excellent

The above formulas were employed for selecting appropriate test items, eliminating unsuitable ones, and adjusting test item formats when necessary.

Statistical analysis

The data was analyzed using Microsoft Excel (Microsoft Corp., Redmond, WA) to perform descriptive statistics such as percentages, frequency, mean, and standard deviation (SD). The results are displayed in tables and bar charts.

## Results

A total of 148 students participated in the examination, which consisted of 50 test items. The test results ranged from 21 to 45, with a mean (SD) score of 35.41 (6.57).

Table 3 presents the mean (SD) values for the two parameters concerning the item analysis of objective questions. For the T/F statements, the DIF I and DI were 76.17 (20.42) and 0.18 (0.13), respectively. Similarly, the DIF I and DI for the fill-in-the-blank statements were also found to be 64.80 (20.70) and 0.32 (0.18), respectively.

**Table 3:** Mean values of various indices used in item analysis N = total number of test items; n = number of true/false statements and fill-in-the-blank statements

Types of items (N = 50)	Difficulty Index	Discrimination Index
T/F statement (n = 30)	76.17 ± 20.42	0.18 ± 0.13
Fill in the blank (n = 20)	64.80 ± 20.70	0.32 ± 0.18

The results for the DIF I revealed that a majority of test items, 60% of the true/false (T/F) statements and 50% of fill-in-the-blank questions, were classified as "too easy." Additionally, 40% of test items were deemed to have a good level of difficulty. However, 10% of fill-in-the-blank statements were considered "too hard." None of the T/F items were categorized as "too difficult" (Figure 1).

**Figure 1:**
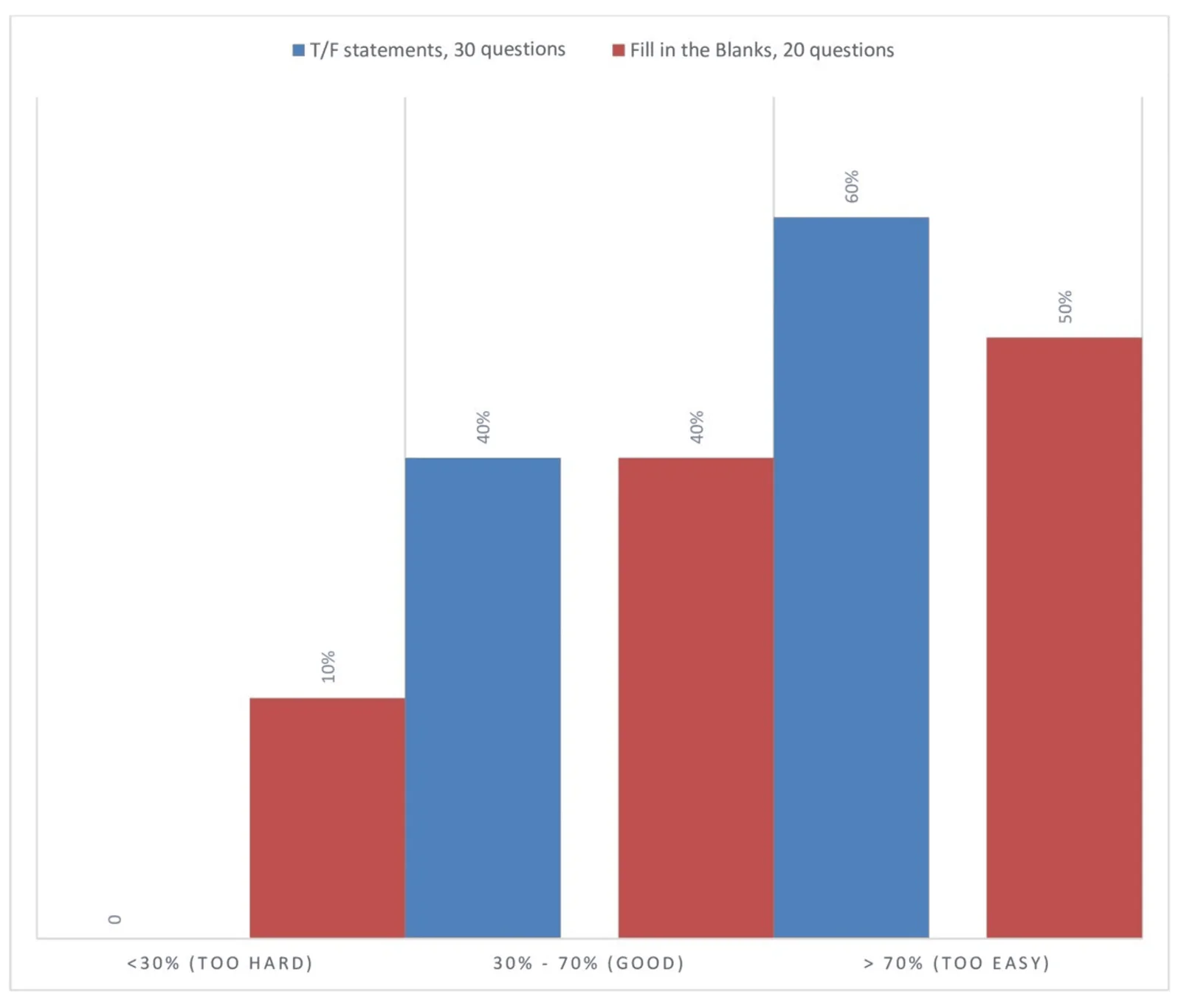
Percentage distribution of items according to their Difficulty Index T/F: true/false

Half the T/F statements had a low Discrimination Index with DI < 0.20. As for the 20 fill-in-the-blank statements, most of these items (70%) were discriminated as either acceptable (5%), good (25%), or excellent (45%) (Figure 2).

**Figure 2:**
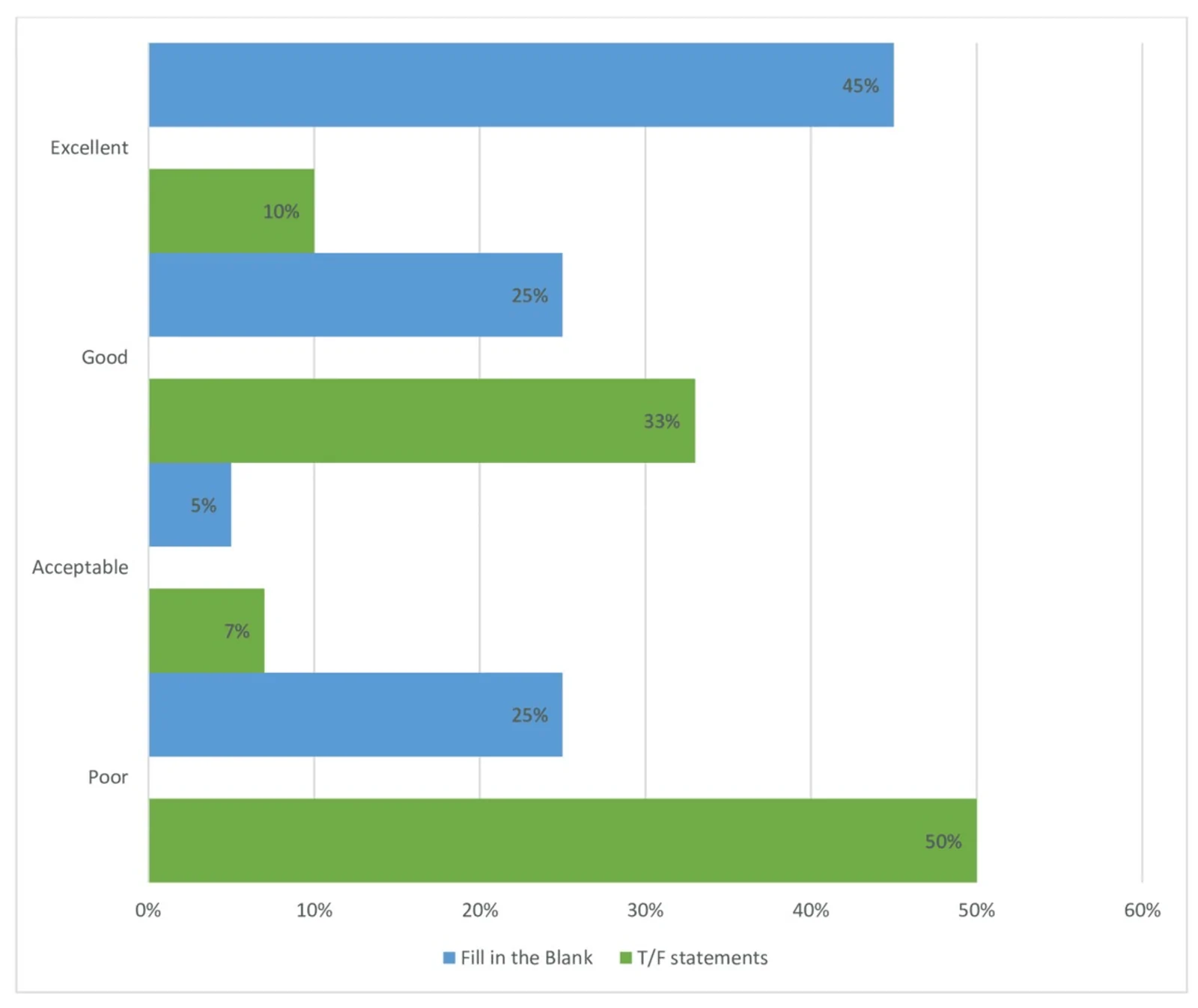
Percentage distribution of items according to their Discrimination Index T/F: true/false

## Discussion

Emphasizing the importance of assessment in medical education is essential. A carefully designed assessment allows educators to evaluate advanced cognitive abilities within the learning process [22]. Moreover, it plays a critical role in underscoring the necessity of evaluating the quality and performance of items after administering an assessment tool. Such evaluation serves as a foundation for improving these items for future utilization, informed by the insights obtained through the review process.

Well-structured test items serve as a potent assessment instrument. However, their efficacy depends entirely on the quality of objective elements that can be evaluated through posttest "item analysis" [16]. The inclusion of items in an assessment requires the careful scrutiny of each item's difficulty level and discriminative ability.

The mean DIF I of T/F statements and fill-in-the-blank statements observed in this study was 76.17 ± 20.42 and 64.80 ± 20.70, respectively. This indicates that, on average, the T/F statements were relatively easy for the students to attempt. Notably, the acceptable range for the DIF I lies between 30% and 70%. So, the fill-in-the-blank statements had the appropriate level of difficulty.

Regarding the distribution of items according to their Difficulty Index, approximately 40% of the items within this study fell within the acceptable difficulty range. However, a larger proportion, between 50% and 60%, was categorized as "too easy." This finding contrasts with previous research, where a majority of questions often fell within the acceptable difficulty levels [23,24]. For instance, a study highlighted that a significant 78% of its items aligned with the acceptable difficulty range [17]. Conversely, a study by Shete et al. found that a mere 30% of their items satisfied the criteria for the acceptable range [18].

The analysis extended to evaluating the discrimination capability of test items, which is crucial for distinguishing between high-ability students (those who have mastered the content) and low-ability students (those who have not achieved mastery). The mean DI of the T/F statements was 0.18 ± 0.13, with 50% of the true/false statements exhibiting a low Discrimination Index, with a DI of <0.20. This result aligns with the findings reported by Gajjar et al., highlighting a common challenge in achieving high discriminative power in assessment items [16].

The DI of the fill-in-the-blank statements was found to be 0.32 ± 0.18, indicating that 75% of these items demonstrated a quality ranging from acceptable/good to excellent. Kolte reported that most (60%) of items exhibited excellent discriminative capability, while a further 17.5% demonstrated good discriminative power, and 20% displayed acceptable discriminative power [19]. These results suggest that these items are well-constructed and do not require modifications. Items categorized as acceptable, good, or excellent are suitable for reuse and can be stored in the question bank for future assessments.

Conversely, five fill-in-the-blank statements exhibited a low Discrimination Index, with DI < 0.20. A value of less than 0.2 suggests inadequate discriminatory capability, suggesting that these items are ineffective at differentiating between higher- and lower-ability students. Moreover, a negative DI value indicates a problematic item, often answered correctly by underperforming students, likely due to random guessing. A study revealed that almost 66% of the items having poor discrimination required significant revisions or complete removal from the test, indicating a potential lack of quality in the test items [21].

A limitation of this study is that a good question needs to go through several levels of screening by peers who are content experts. In this study, due to a lack of content experts, only the course coordinator selected the test items, which were then finalized for the examination. Additionally, this was a single-center study because the course was held only at BSMMU, where most Phase B residents took part. The main goal of this study was to evaluate the objective test items and check their quality for building a question bank. As a result, only limited psychometric analysis was carried out; internal-consistency reliability measures (e.g., Cronbach's alpha or KR-20) were not computed in this study, and future work should incorporate such reliability analysis alongside item-level indices for a more complete evaluation of test quality. Furthermore, the item pool was relatively small (50 items in total, comprising 30 T/F and 20 fill-in-the-blank statements), which limits the precision of the difficulty and discrimination estimates and constrains the generalization of these findings to a larger question bank.

## Conclusions

The study found that half of the test items had a good Difficulty Index, indicating their suitability for residents. Two-thirds of fill-in-the-gap statements had adequate discrimination power, indicating no immediate revision or replacement. However, 50% of the T/F statements had a low Discrimination Index, indicating their revision or rejection. The study suggests that future iterations of this course incorporate structured content-expert review as a preceding step to item-level statistical analysis, and the test items need further refinement to improve their quality for future assessments.
